# Involvement of a specificity proteins-binding element in regulation of basal and estrogen-induced transcription activity of the *BRCA1 *gene

**DOI:** 10.1186/bcr1987

**Published:** 2008-03-31

**Authors:** Jennifer K Hockings, Stephanie C Degner, Sherif S Morgan, Michael Q Kemp, Donato F Romagnolo

**Affiliations:** 1 Cancer Biology Interdisciplinary Graduate Program, Department of Nutritional Sciences, The University of Arizona, E 4th Street, Tucson, Arizona 85721-0038, USA; 2 Laboratory of Mammary Gland Biology, Department of Nutritional Sciences, The University of Arizona, E 4th Street, Tucson, Arizona 85721-0038, USA

## Abstract

**Introduction:**

Increased estrogen level has been regarded to be a risk factor for breast cancer. However, estrogen has also been shown to induce the expression of the tumor suppressor gene, *BRCA1*. Upregulation of BRCA1 is thought to be a feedback mechanism for controlling DNA repair in proliferating cells. Estrogens enhance transcription of target genes by stimulating the association of the estrogen receptor (ER) and related coactivators to estrogen response elements or to transcription complexes formed at activator protein (AP)-1 or specificity protein (Sp)-binding sites. Interestingly, the BRCA1 gene lacks a consensus estrogen response element. We previously reported that estrogen stimulated BRCA1 transcription through the recruitment of a p300/ER-α complex to an AP-1 site harbored in the proximal BRCA1 promoter. The purpose of the study was to analyze the contribution of *cis*-acting sites flanking the AP-1 element to basal and estrogen-dependent regulation of BRCA1 transcription.

**Methods:**

Using transfection studies with wild-type and mutated BRCA1 promoter constructs, electromobility binding and shift assays, and DNA-protein interaction and chromatin immunoprecipitation assays, we investigated the role of Sp-binding sites and cAMP response element (CRE)-binding sites harbored in the proximal BRCA1 promoter.

**Results:**

We report that in the BRCA1 promoter the AP-1 site is flanked upstream by an element (5'-GGGGCGGAA-3') that recruits Sp1, Sp3, and Sp4 factors, and downstream by a half CRE-binding motif (5'-CGTAA-3') that binds CRE-binding protein. In ER-α-positive MCF-7 cells and ER-α-negative Hela cells expressing exogenous ER-α, mutation of the Sp-binding site interfered with basal and estrogen-induced BRCA1 transcription. Conversely, mutation of the CRE-binding element reduced basal BRCA1 promoter activity but did not prevent estrogen activation. In combination with the AP-1/CRE sites, the Sp-binding domain enhanced the recruitment of nuclear proteins to the BRCA1 promoter. Finally, we report that the MEK1 (mitogen-activated protein kinase kinase-1) inhibitor PD98059 attenuated the recruitment of Sp1 and phosphorylated ER-α, respectively, to the Sp and AP-1 binding element.

**Conclusion:**

These cumulative findings suggest that the proximal BRCA1 promoter segment comprises *cis*-acting elements that are targeted by Sp-binding and CRE-binding proteins that contribute to regulation of BRCA1 transcription.

## Introduction

Gene expression in mammary tissue is under the control of ovarian steroids, including estrogen, which regulates transcription activity of target promoters by stimulating the recruitment of either the estrogen receptor (ER)-α or ER-β [1]. In the classical pathway, the liganded ER-α homodimerizes and binds directly to DNA at estrogen response elements (EREs) [2]. The physical contact of the ER-α homodimer with DNA facilitates the subsequent recruitment of coregulators [3]. The latter factors contribute to chromatin reorganization and transcriptional coactivation [4]. Alternatively, the ER-α can physically interact with DNA-bound protein complexes containing, among other factors, the activator protein (AP)-1 [5], cAMP response element (CRE [TGACGTCA])-binding protein (CREB) [1], or specificity protein (Sp) family members [6,7]. The Sp factors have been shown to bind directly to the consensus GC-rich region 5'-(G/T)GGGCGG(G/A)(G/A)-3' or DNA-bound AP-1 transcription complexes [8].

Members of the Sp family of transcription factors exert differential effects on gene transcription. For example, Sp1 and Sp4 have been shown to act as transcriptional activators, whereas Sp3 was reported to antagonize Sp1 activation functions by competing for promoter occupancy [9-11]. The Sp family members play a significant role in tissue and organ development, because mice lacking at least one of the Sp proteins exhibit various physiological abnormalities, including embryonic lethality [12], growth retardation [13,14], and sterility [15]. Increased expression of Sp1 has been detected in gastric [16,17] pancreatic [18], and breast [19] tumors. In addition to inducing the recruitment of ER to target promoters, estrogen influences gene expression through the activation of mitogen-activated protein kinases (MAPKs), which phosphorylate the ER-α and other transcription factors including Sp1 [20] and AP-1 [2]. Phosphorylation of these factors increases their binding affinity for cognate DNA elements or DNA-bound coregulators [21].

The breast cancer susceptibility gene *BRCA1 *encodes a phosphoprotein that is involved in the DNA damage response [22,23] and regulation of cell cycle checkpoints [24]. About 30% to 40% of sporadic breast cancers, which represent the vast majority (90% to 95%) of breast cancer cases, exhibit lower or absent levels of BRCA1 in the absence of mutations in the *BRCA1 *gene [25,26]. Because altered regulation of *BRCA1 *may increase the likelihood of developing breast cancer, it is important to examine the molecular events that regulate normal BRCA1 expression.

In proliferating breast epithelial cells, the cellular levels of BRCA1 mRNA and protein have been shown to increase in response to estrogen [27-29]. The *BRCA1 *promoter harbors two distinct transcriptional start sites (exon-1A and exon-1B). However, translation of *BRCA1 *mRNA always starts from the ATG codon located on exon-2 [30]. Upregulation of BRCA1 expression in response to estrogen may represent a feedback mechanism that represses ER-signaling during the early stages of breast tumorigenesis [31]. This interpretation is supported by evidence indicating that the BRCA1 protein represses the transcriptional activity of the liganded ER-α and estrogen-regulated genes [32]. However, the *BRCA1 *gene appears not to be regulated by estrogen through the classical pathway, because the *BRCA1 *promoter lacks canonical EREs. Recently, our laboratory reported that estrogen stimulated *BRCA1 *promoter activity by inducing the recruitment of an ER-α/p300 transcription complex to an AP-1 element in close proximity to the transcriptional start site of exon-1B [33,34]. The objective of the present work was to elucidate the roles played by Sp-binding and CRE-binding elements flanking the AP-1 site in regulation of BRCA1 transcription.

## Materials and methods

The experimental research reported in the manuscript has been performed with the approval of the Institutional Biosafety Committee of the University of Arizona (Tucson, AZ, USA), on 20 December 2005.

### Cell culture and chemicals

MCF-7 and HeLa cells were obtained from the American Type Culture Collection (Manassas, VA, USA) and maintained in Dulbecco's modified Eagle's medium (DMEM)/F12 (Sigma-Aldrich Chemical Co., St. Louis, MO, USA), supplemented with 10% fetal bovine serum (FBS; Hyclone Laboratories, Logan, UT, USA) as described previously [29]. Estrogen, penicillin/streptomycin solution, and DMEM/F12 were purchased from Sigma-Aldrich Chemical Co. Antibodies against ER-α, Sp1, Sp3, and Sp4 were purchased from Santa Cruz Biotechnologies, Inc. (Santa Cruz, CA, USA). The antibody against phosphorylated ER-α (Ser118) was obtained from Cell Signaling (Beverly, MA, USA).

### Site-directed mutagenesis

Details concerning the cloning of a 1.7-kilobase *BRCA1 *promoter fragment into pGL3 Basic (pGL3BRCA1) are described elsewhere [35]. Mutation of the Sp (GGGGCGG to GCTAAG) and half-CRE (CGTAA to CtgcA) core sequence in the *BRCA1 *promoter was carried out by site-directed mutagenesis (Stragene, La Jolla, CA, USA) using the following primers synthesized by Sigma-Genosys (The Woodlands, TX, USA): Sp-F-Mut: 5'-GGGTA**GGctaaGAA**CCTGAGAGGCGTAAGG CG-3'; Sp-R-Mut: 5'-CGCCTTACGCCTCTCA GG**TTCttagCC**TACCC-3'; CRE-F-Mut: 5'-GGAACCTGAGAGG**CtgcA**GGCGTTGTGAAG-3'; and CRE-R-Mut: 5'-CTTCACAACGCC**TgcaG**CCTCTCAGGTTCC-3'. The insertion of mutations was confirmed by direct sequencing.

### Transfection studies

Experimental conditions for cell culture and transfection of reporter plasmids were those described previously [33]. Briefly, MCF-7 and HeLa cells were cultured for 3 days in phenol red-free DMEM/F12 and supplemented with 5% charcoal-stripped FBS. Then, cells were seeded in six-well plates 24 hours before transfection using the Lipofectamine-Plus (Invitrogen, Carlsbad, CA, USA). Plasmids encoding renilla were also cotransfected to account for variations in transfection efficiency and cell density. Luciferase reporter activity was monitored with a Luminometer 20/20 (Turner Biosystems, Sunnyvale, CA, USA) and expressed as relative luciferase units corrected for renilla.

### DNA-protein pull-down assay

Eighty to ninety per cent confluent breast MCF-7 cells were cultured for 3 days in phenol red-free DMEM/F12 and supplemented with 5% charcoal-stripped FBS. Cells were then maintained in phenol red-free DMEM/F12 plus vehicle with 5% charcoal-stripped FBS with or without 10 nmol/l estrogen before nuclear extracts were obtained using the Nuclear/Cytoplasmic Extract Kit and quantitated using the BCA protein assay (Pierce Biotechnology, Rockford, IL, USA). The biotin-labeled double stranded oligonucleotides were based on the human *BRCA1 *promoter sequence. The DNA-protein pull-down assay was performed as described previously [36]. Briefly, MCF-7 nuclear extracts were mixed with biotin-labeled oligonucleotides and streptavidin agarose beads with 70% slurry. After 2 hours of incubation at room temperature, beads were centrifuged and washed with cold phosphate buffer saline. Bound proteins were then separated on 4% to 12% PAGE and Western blot analysis was performed with antibodies against Sp1, Sp3, total ER-α, or phosphorylated ER-α. Oligonucleotides used for the pull-down assay were as follows: Sp/AP-1, forward: 5'-GGGTA**GGGGCGGAA**CCTGAGAGGCGTAA-3'; Sp/AP-1, reverse: 5'-TTACGCCTCTCAGG**TTCCGCCCC**TACCC-3'; Sp-Mut/AP-1, forward: 5'-GGGTA**GGctaaGAA**CCTGAGAGGCGTAA-3'; and Sp-Mut/AP-1, reverse: 5'-TTACGCCTCTCAGG**TTCttagCC**TACCC-3'.

### Electrophoretic mobility binding and shift assays

The procedure used for electromobility binding assay was described previously [33]. Cells were plated in 6-well Costar tissue culture plates in DMEM plus 5% charcoal-stripped FBS. After 24 hours, cells were cultured in control DMEM plus vehicle or 10 nmol/l estrogen. Then, cells were trypsinized and washed with ice-cold phosphate buffer saline. Cells were resuspended in ice-cold 25 mmol/l Hepes buffer containing 1.5 mmol/l EDTA, 1 mmol/l DTT, 0.5 mmol/l PMSF and 5 μg/ml aprotinin, and placed on ice for 10 minutes. Cells were pelleted and resuspended in 1 ml ice-cold 25 mmol/l Hepes buffer containing 1.5 mmol/l EDTA, 10% (vol/vol) glycerol, 1 mmol/l DTT, 0.5 mmol/l PMSF, and 5 μg/ml aprotinin. The cell suspension was transferred to a mortar for drilling with a Teflon pestle until more than 90% of the cells in a 2 μl aliquot were unable to exclude trypan blue. After centrifugation, cell pellets were resuspended in 150 μl ice-cold 25 mmol/l Hepes buffer containing 1.5 mmol/l EDTA, 10% (vol/vol) glycerol, 0.5 mol/l KCl, 1 mmol/l DTT, 0.5 mmol/L PMSF and 5 μg/ml aprotinin, and placed on ice with intermittent vortexing. Cell debris was removed by centrifugation. Supernatants containing nuclear protein were stored at -70°C. Nuclear protein concentration was determined using the BCA protein assay (Pierce Chemical Company). Oligonucleotides used for binding and shift assays are summarized in Table 1. The complementary oligonucleotides were annealed then phosphorylated at the 5'-end with [γ-^32 ^P]ATP and T4 polynucleotide kinase. Unincorporated nucleotides were removed using the TE-10 spin columns (Clontech Laboratories Inc., Mountain View, CA, USA). Binding assays were performed by incubating 5 μg nuclear protein in the binding buffer then incubated with the labeled oligonucleotides for 20 minutes. For supershift assays, antibodies (Affinity Bioreagents, Golden, CO, USA) were incubated with 1 μg nuclear extracts for 2 hours before addition of labeled oligonucleotides. For cold competition, a 100-fold excess of the respective unlabeled oligonucleotides was added to the binding reaction 10 minutes before addition of the labeled oligonucleotides. For supershift assays, antibodies (Santa Cruz Biotechnologies) were incubated with 1 μg nuclear extracts for 2 hours before addition of labeled oligonucleotides. For cold competition, a 100-fold excess of the respective unlabeled oligonucleotides was added to the binding reaction 10 minutes before addition of the labeled oligonucleotides. Samples were electrophoresed through a 5% nondenaturing polyacrylamide gel at 200 V for 90 minutes. Finally, the gel was dried and exposed to a phosphor screen, and digital phosphorimages were retrieved using the Storm system (Molecular Dynamics, Sunnyvale, CA, USA).

**Table 1:** BRCA-1 Oligonucleotides used for binding and shift assays

Promoter element	Oligonucleotides
AP-1/CRE	Sense: 5'-AAC**CTGAG**AGG**CGTAA**GGCGT-3',
	Antisense: 5'-ACGCC**TTACG**CCT**CTCAG**GTT-3';
Sp/AP-1/CRE	Sense: 5'-GGGTA**GGGGCGGAA**C**CTGAG**AGG**CGTAA**GGCGT-3',
	Antisense: 5'-ACGCC**TTACG**CCT**CTCAG**G**TTCCGCCCC**TACCC-3';
Sp-Mut/AP-1/CRE	Sense: 5'-GGGTA**GGctaaG**AACCTGAGAGGCGTAAGGCGT-3',
	Antisense: 5'-ACGCCTTACGCCTCTCAGGTT**CttagCC**TACCC-3'
Sp/AP-1-Mut/CRE	Sense: 5'-GGGTAGGGGCGGAAC**tatAG**AGGCGTAAGGCGT-3',
	Antisense: 5'-ACGCCTTACGCCT**CTata**GTTCCGCCCCTACCC-3';
	Sense: 5'-GGGTAGGGGCGGAAC**Cacta**AGGCGTAAGGCGT-3',
	Antisense: 5'-ACGCCTTACGCCT**tagtG**GTTCCGCCCCTACCC-3';
Intervening AP-1/CRE sequences:	Sense: 5'-GGGTAGGGGCGGAACCTGA**tct**GCGTAAGGCGT-3',
	Antisense: 5'-ACGCCTTACGC**aga**TCAGGTTCCGCCCCTACCC-3';
	Sense: 5'-GGGTAGGGGCGGAACCTGAGA**tta**GTAAGGCGT-3',
	Antisense: 5'-ACGCCTTAC**taa**TCTCAGGTTCCGCCCCTACCC-3';
Sp/Ap-1/CRE-Mut	Sense: 5'-GGGTAGGGGCGGAACCTGAGAGG**CtgcA**GGCGT-3',
	Antisense: 5'-ACGCC**TgcaG**CCTCTCAGGTTCCGCCCCTACCC-3';
	Sense: 5'-GGGTAGGGGCGGAACCTGAGAGG**CGTcttG**CGT-3',
	Antisense: 5'-ACGC**aagACG**CCTCTCAGGTTCCGCCCCTACCC-3';

AP, activator protein; CRE, cAMP response element; Sp, specificity protein.

### Chromatin immunoprecipitation assay and real-time PCR

Chromatin immunoprecipitation assay and real-time PCR were carried out as described previously [34,37]. Briefly, MCF-7 cells were prepared in phenol red-free DMEM/F12 supplemented with 5% charcoal-stripped FBS for 3 days. After treatment with control DMEM plus vehicle or 10 nmol/l estrogen, chromatin was crosslinked with formaldehyde (1% final concentration) for 10 minutes at room temperature and subsequently glycine was added to quench the formaldehyde crosslinking. Cells were then harvested and resuspended in lysis buffer (1% SDS, 10 mmol/l EDTA, 50 mmol/l Tris-HCl, and protease inhibitor cocktail). After sonication (10 × 15 seconds), samples were diluted in chromatin immunoprecipitation (ChIP) buffer (1% Triton X-100, 2 mmol/l EDTA, 150 mmol/l NaCl, 20 mmol/l Tris-HCl, and protease inhibitor cocktail). Dilutions of chromatin preparations were reserved as either input (no antibody) or used for immunoprecipitation with the desired antibody or IgG as a negative control. The chromatin immunoprecipitation was conducted utilizing the Magna ChIP kit (17–611) obtained from Millipore (Billenca, MA, USA), in accordance with the protocol provided. Briefly, the sonicated samples were immunoprecipitated overnight at 4°C with the antibodies and G magnetic beads. The magnet beads were pelleted and sequentially washed with a low salt immune complex wash buffer, high salt immune complex wash buffer, LiCl immune complex wash buffer, and TE buffer. The protein-DNA complexes were eluted and crosslinking was reversed by incubation with ChIP elution buffer and proteinase K for 2 h at 62°C, followed by incubation for 10 minutes at 95°C. The free DNA was then purified using the provided spin columns.

PCR primers used to amplify the *BRCA1 *promoter region flanking the Sp/AP-1/CRE binding sites were as follows: forward: 5'-CTGACAGATGGGTATTCTTTGACG-3'; and reverse: 5'-GCATATTCCAGTTCC TATCACGAG-3' (171 base pairs). The iTaq SYBR Green Supermix reagents (Bio-Rad, Hercules, CA, USA) were used as described by the manufacturer. Reactions were run at a final volume of 25 μl consisting of 12.5 μl of 2× SYBR Green Supermix with ROX (0.4 mmol/l each of dATP, dCTP, dGTP, and dTTP, 50 U/ml iTaq DNA polymerase, 6 mmol/l Mg^2+^, SYBR Green I, and ROX reference dye), 1 μl each of forward and reverse primers added at a final concentration of 200 nM, 2 μl DNA, and 8.5 μl nuclease-free double-distilled water. The ABI 5700 sequence detection system and comparative C_T _method were used to quantify the relative differences in PCR. *BRCA1 *promoter amplicons were normalized to input.

### Statistical analysis

Results of transfection studies and real-time PCR from ChIP assays are presented as means ± standard error of the mean. Statview, the SAS Institute (Cary, NC, USA) statistical analysis software was used for analysis of variance. Comparison of means following a significant (*P *< 0.05) analysis of variance test was performed by Fisher's protected least significant difference test.

## Results

**Figure 1:**
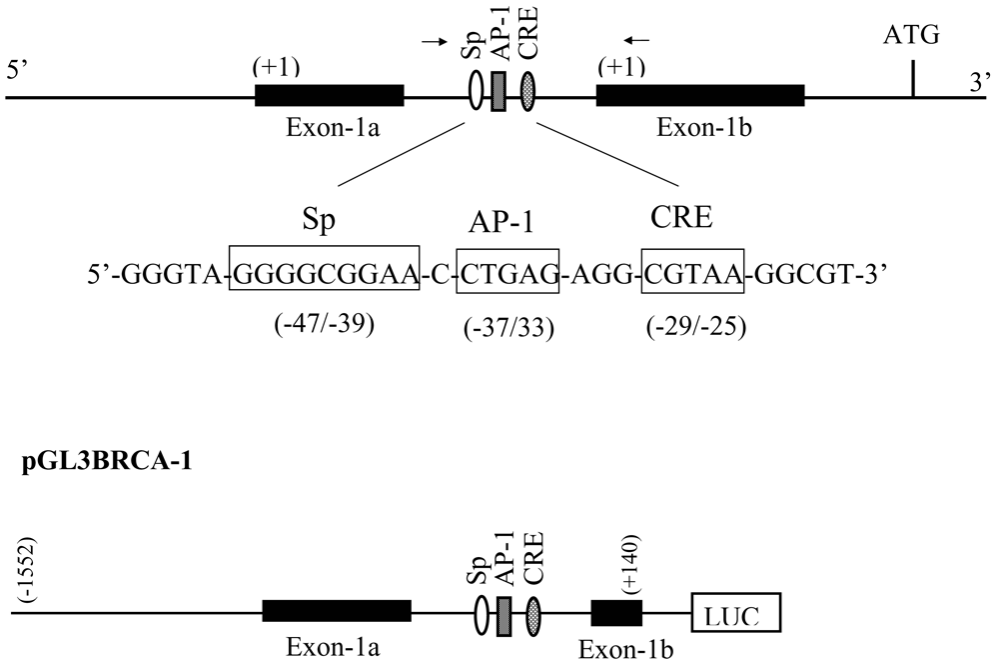
Regulatory sites of the human *BRCA1 *promoter. A specificity protein (Sp) consensus site located upstream of an activator protein (AP)-1 element in the intervening sequence between exon-1a and exon-1b contributes to basal and estrogen-regulated transcription. A cAMP response element (CRE) site located downstream of the AP-1 contributes to basal regulation. Numbers in parenthesis for the Sp, AP-1, and CRE elements are base pairs from the initiation of transcription (+1) on exon-1b. Nucleotides in boxes represent the sequence and spatial arrangement of the Sp, AP-1, and CRE domains. Arrows flanking the Sp/AP-1/CRE sites indicate the position of oligonucleotides used for real-time amplification after chromatin immunoprecipitation assays. The specific sequences of oligonucleotides are reported in the Materials and methods section. pGL3BRCA-1 represents a 1.692 kilobase BRCA1 promoter-luciferase (LUC) construct used in transfection studies. This promoter fragment extends from +140 base pairs to – 1.552 kilobases from the +1 of exon-1b. Details of the recruitment of a p300/ER-α/SRC-1 complex to the AP-1 site are described elsewhere [33].

The primary objective of this study was to examine whether *cis*-acting elements flanking an AP-1 site located in the proximal *BRCA1 *promoter played a role in the basal and estrogen-dependent regulation of BRCA1 transcription. We mapped immediately upstream from the AP-1 site a GC-rich region located at positions -41/-36 (GGGGCGGAA) that shared perfect homology to the consensus Sp-binding sequence (5'-[G/T]GGGCGG [G/A] [G/A]-3') [8] (Figure 1). We also mapped a half-CRE-binding site (5'-CGTAA-3') immediately downstream (-23/-27) from the AP-1 element. Previous investigations from our laboratory have documented that estrogen-dependent stimulation of BRCA1 transcription required the assembly of an ER-α/p300 complex at the AP-1 site [33]. Because Sp-binding factors and CRE-binding factors have been shown to cooperate with AP-1 in regulation of transcription, we examined the role played by these sites in controlling basal and estrogen-induced *BRCA1 *promoter activity. Breast cancer MCF-7 cells were transiently transfected with either a reporter-luciferase construct containing 1.7 kilobase of the wild-type *BRCA1 *promoter (pGL3BRCA1, -1552 to +140 from +1 on exon-1b) or the pGL3BRCA1 construct carrying mutated Sp (pSpmut, 5'-GGctaaGAA-3') or CRE (pCREmut, 5'-CtgcA-3').

**Figure 2:**
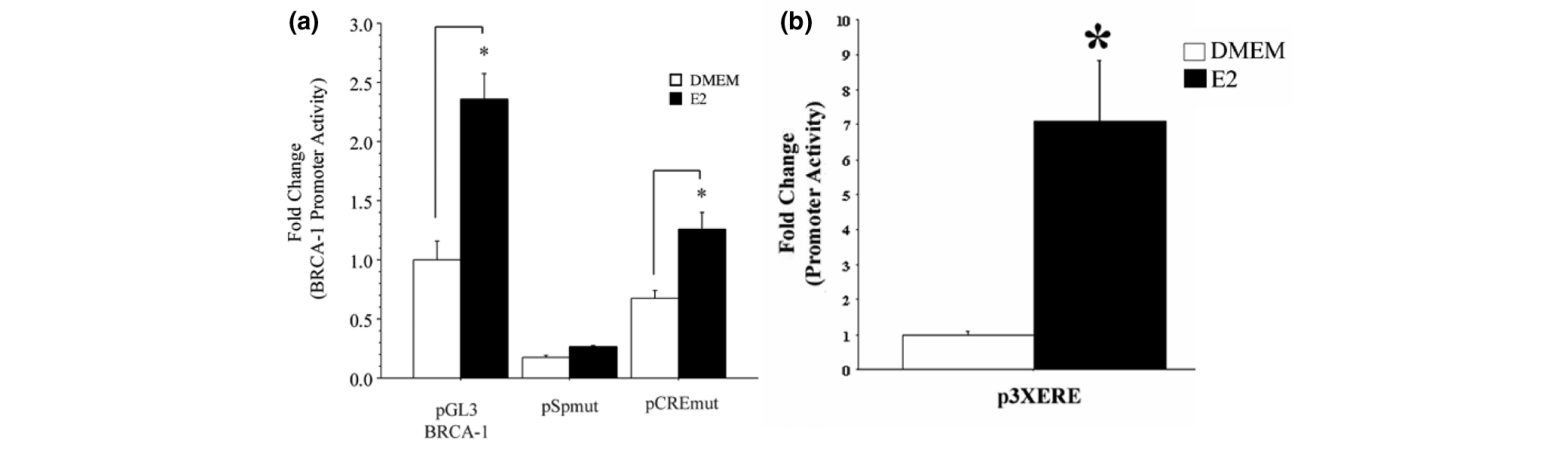
Sp site contributes to estrogen-dependent activation of the *BRCA1 *promoter in breast cancer MCF-7 cells. **(a) **Cells were precultured for 3 days in phenol red-free Dulbecco's modified Eagle's medium containing 5% charcoal dextran-stripped fetal bovine serum (DMEM). Then, MCF-7 cells were transiently transfected with pGL3BRCA-1, pSpmut, or pCREmut. Cells were cultured in control vehicle (DMEM) or DMEM plus 10 nmol/l 17β-estradiol (E2) for 24 hours. **(b) **The treatment with E2 induced promoter activity from a positive control vector (p3xERE) transfected into MCF-7 cells. Bars represent mean luciferase units corrected for the internal control renilla ± standard error from three independent experiments performed in quadruplicate. Asterisks indicate significant E2 activation (*P < 0.05) of promoter activity as compared with vehicle control (DMEM) in cells transfected with pGL3BRCA-1, pSpmut, or pCREmut.

In MCF-7 cells transfected with pGL3-BRCA1, the treatment with estrogen induced (about 2.3-fold) *BRCA1 *promoter activity (Figure 2a) as compared with the control vehicle (DMEM), thus confirming the results of previous findings from our laboratory [34]. However, basal reporter activity was reduced by about 80% in MCF-7 cells transfected with pSpmut. We observed a small increase (1.2-fold; *P *< 0.10) in *BRCA1 *promoter activity upon treatment with estrogen, but this stimulation was considerably lower than that observed in cells transfected with the wild-type pGL3 *BRCA1 *promoter construct. Conversely, the mutation of the half CRE-binding site reduced by about 35% basal *BRCA1 *promoter activity but did not interfere with estrogen-dependent activation (about 2.0-fold) of BRCA1 transcription. These results clearly highlighted the differential role of the Sp site compared with the half-CRE in the estrogen-dependent regulation of BRCA1. The efficacy of the transfection conditions and estrogen treatment were confirmed by the activation of transcription from a positive control promoter-luciferase construct harboring three consensus EREs (p3XERE; Figure 2b).

**Figure 3:**
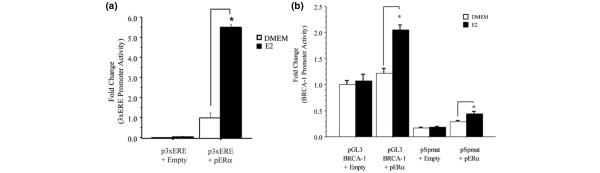
Sp site contributes to E2-dependent activation of the *BRCA1 *promoter in Hela cells expressing exogenous ER-α. **(a) **Hela cells were cotransfected with pGL3BRCA-1 or pSpmut containing a mutated specificity protein (Sp) site plus the empty vector pCR3.1 or pERα. Transfected cells were cultured in Dulbecco's modified Eagle's medium containing 5% charcoal dextran-stripped fetal bovine serum plus vehicle (DMEM) or DMEM plus 10 nmol/l 17β-estradiol (E2) for 24 hours. **(b) **Hela cells were cotransfected with an empty plasmid (PCR3.1) plus p3xERE or p3XERE plus a vector containing the cDNA for the estrogen receptor (ER)-α (pERα). Bars represent mean luciferase units corrected for the internal control renilla ± standard error from four independent experiments performed in quadruplicate. Asterisks indicate significant E2 activation (*P < 0.05) of *BRCA1 *promoter activity as compared with vehicle control (DMEM).

Based on this information, we analyzed the cooperativity between the Sp site and ER-α in estrogen-activation of the BRCA1 promoter. For these experiments, we selected the Hela cell line because it does not express ER-α and in previous studies [34], we successfully used this system to examine estrogen-regulated BRCA1 expression. In preliminary experiments, we ascertained that the cotransfection of Hela cells with p3XERE plus an empty vector (pCR3.1) did not yield measurable luciferase activity (Figure 3a). Compared with control vehicle (DMEM), the treatment with estrogen stimulated luciferase reporter activity in Hela cells cotransfected with a vector containing the cDNA cassette for ER-α (pERα) plus p3XERE. These results confirmed the validity of the Hela cell line as a model system to study the regulation of BRCA1 by estrogen. In Hela cells transfected with pGL3BRCA1 plus the empty vector pCR3.1, the treatment with estrogen did not induce *BRCA1 *promoter activity, although it was stimulated about 2.0-fold upon cotransfection with pERα (Figure 3b). In Hela cells cotransfected with the pSpmut construct alone, the basal promoter activity was reduced by 80%, and estrogen activation was reduced in magnitude from 2.0-fold to 1.4-fold (*P *< 0.05). These results confirmed the important role of the crosstalk between the Sp site and ER-α in basal and estrogen-dependent regulation of BRCA1 transcription.

**Figure 4:**
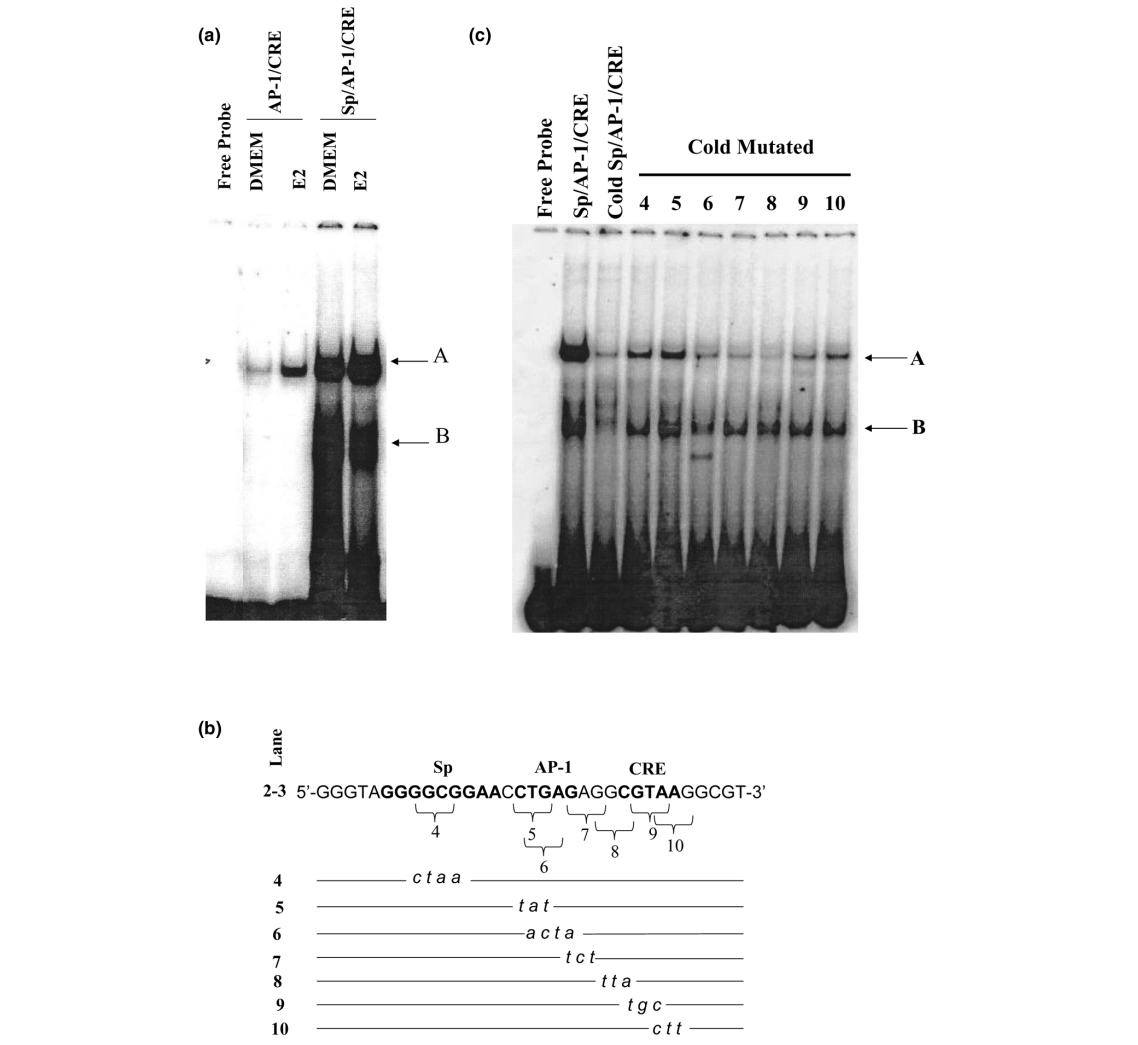
Sp site enhances the binding of nuclear proteins to the *BRCA1 *promoter. **(a) **Nuclear extracts normalized to 5 μg of nuclear protein obtained from MCF-7 cells were incubated with ^32^P-labeled *BRCA1 *promoter oligonucleotides containing the activator protein (AP)-1/cAMP response element (CRE) or the Sp plus AP-1/CRE sites. MCF-7 cells were cultured for 3 hour in control vehicle (DMEM) or DMEM plus 10 nmol/l 17β-estradiol (E2). FP, free probe (lane 1). **(b, c) **In competition studies, nuclear extracts from MCF-7 cells cultured in the presence of E2 were incubated with ^32^P-labeled BRCA1 oligonucleotides containing the wild-type specificity protein (Sp)/AP-1/CRE sequences in the absence (lane 2) or presence (100-fold) of competing cold wild-type Sp/AP-1/CRE oligonucleotide (lane 3), or 100-fold of competing cold oligonucleotides mutated for Sp (lane 4), AP-1 (lanes 5 and 6), intervening sequences between AP-1 and CRE (lanes 7 and 8), and CRE (lanes 9 and 10). The arrows indicate the DNA:protein complexes visualized by electromobility binding assay.

Next, we examined whether the Sp sequence influenced the recruitment of nuclear factors to the adjacent region of the *BRCA1 *promoter harboring the AP-1 and CRE sites. BRCA1 oligonucleotides were coincubated with nuclear extracts obtained from MCF-7 cells cultured in control vehicle (DMEM) or in the presence of estrogen. The results of electromobility binding assays indicated that estrogen stimulated the binding of nuclear factors to the BRCA1 oligonucleotide harboring the AP-1 and CRE sites as evidenced by the increase in intensity of a DNA:protein complex (complex A; Figure 4a). However, we observed a striking increase in the intensity of this complex when nuclear extracts were coincubated with the BRCA1 oligonucleotide spanning the Sp-, AP-1, and CRE domains. Moreover, a second complex (complex B) of lower molecular weight was visualized by gel electrophoresis after incubation of nuclear extracts with the Sp/AP-1/CRE oligonucleotide. These results suggested that the complex B probably comprised Sp proteins.

The role played by the Sp, AP-1, and CRE sites in the recruitment of nuclear factors was further investigated in competition studies with oligonucleotides harboring mutations for each site (Figure 4b). Compared with the wild-type labeled BRCA1 oligonucleotide, the incubation of nuclear extracts with cold wild-type Sp/AP-1/CRE oligonucleotide reduced the binding by nuclear factors (Figure 4c; lane 3). Conversely, the coincubation with cold oligonucleotides harboring mutated Sp, AP-1, or CRE differentially restored binding as compared to the wild-type labeled control (lanes 4 to 10). The most significant recovery of binding was observed with cold oligonucleotides mutated for Sp1 (lane 4), AP-1 (lane 5), and CREB (lanes 9 and 10). These cumulative data confirmed that the Sp, AP-1, and CRE sites played an important role in the formation of transcription complexes at this segment of the *BRCA1 *promoter.

**Figure 5:**
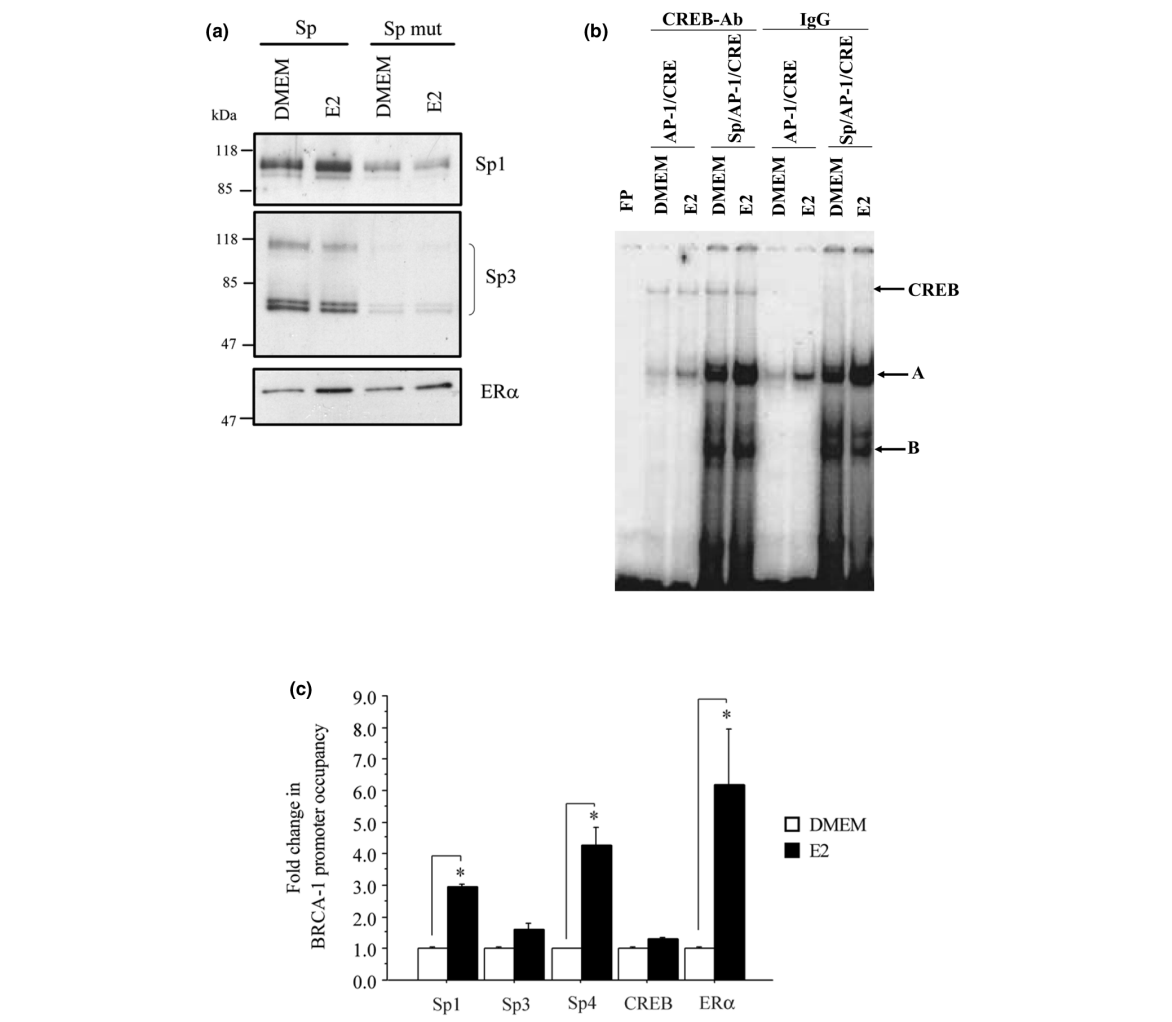
Estrogen stimulates the recruitment of Sp to a GC-rich region in the *BRCA1 *promoter. **(a) **Nuclear protein extracts (200 μg) were obtained from MCF-7 cells cultured for 1.5 hours in Dulbecco's modified Eagle's medium containing 5% charcoal dextran-stripped fetal bovine serum containing vehicle (DMEM) or DMEM plus 10 nmol/l 17β-estradiol (E2). DNA:protein complexes were generated by coincubation of nuclear extracts with the wild-type specificity protein (Sp)1/activator protein (AP)-1 oligonucleotide or the same oligonucleotide harboring a mutated Sp (Spmut) and separated by SDS-PAGE. The recruitment of Sp1, Sp3, or estrogen receptor (ER)-α was visualized by Western blotting with specific antibodies. Panels are representative of two independent experiments. Numbers represent molecular weight markers (kDa). **(b) **Nuclear extracts normalized to 5 μg of nuclear protein obtained from MCF-7 cells were incubated with ^32^P-labeled *BRCA1 *promoter oligonucleotides containing the AP-1/CRE or the Sp plus AP-1/CRE sites. MCF-7 cells were cultured for 3 hours in control vehicle (DMEM) or DMEM plus 10 nmol/l E2. FP, free probe (lane 1). Arrows indicate DNA:protein complexes (A and B) visualized by electromobility binding assay, and cAMP response element (CRE)-binding protein(CREB)/DNA complexes supershifted with the CREB antibody. IgG, preimmune immunoglobulin control. **(c) **Basal and E2-induced recruitment of Sp1, Sp3, Sp4, and ER-α to the *BRCA1 *region containing the Sp, AP-1, and CRE sites was examined by chromatin immunoprecipitation assay followed by real-time PCR, as described in Materials and methods. The columns represent mean amplification products corrected for input ± standard error (bars) and normalized to vehicle control (DMEM). Asterisks indicate statistically significant (*P *< 0.05) induction of factor recruitment by E2 as compared with vehicle control DMEM.

To test whether the GC-box was a binding site for Sp factors and the ER-α, we performed DNA-protein pull-down assays. Nuclear extracts harvested from control (DMEM) and estrogen-treated MCF-7 cells were incubated with BRCA1 oligonucleotides comprising the GC-box. Western blots revealed the constitutive occupancy of Sp-1, Sp3, and ER-α in control vehicle (DMEM) cells (Figure 5a). For the Sp3 protein, we visualized two doublets of approximately 60 and 100 kDa, which represent the two short and two long forms from different translational initiation [38]. A similar pattern for Sp3 has been reported following Western blotting of cellular extracts obtained from MCF-7 cells [39,40]. The treatment with estrogen increased the binding levels of Sp1 and ER-α protein, but not the association of Sp3. The incubation of nuclear extracts with BRCA1 oligonucleotides mutated for the Sp-binding site (Spmut) reduced significantly the binding by Sp1 and Sp3, but minimally reduced the association of ER-α. These results suggested that Sp binding was not required for the recruitment of the ER-α to the adjacent AP-1 site.

Interactions of CREB proteins with the BRCA1 promoter region containing the Sp/AP-1/CRE sequences were investigated by gel electromobility shift assay. Nuclear proteins were incubated with the Sp1/CRE or Sp/Ap1/CRE oligonucleotides and, upon gel separation, we observed (Figure 5b) a pattern of retarded bands (A and B) that was similar to that depicted in Figure 4a. Upon coincubation with an antibody for CREB, we visualized a slower migrating band suggesting this protein was associated with the Sp/CRE or Sp/AP-1/CRE oligonucleotide. These findings suggested that the Sp site or estrogen treatment did not influence the constitutive recruitment of CREB to the AP-1/CRE region.

Further confirmation of the interactions of Sp1, Sp3, Sp4, ER-α, and CREB with the *BRCA1 *promoter was obtained by ChIP assay followed by real-time PCR analysis. The treatment with estrogen increased the occupancy of Sp1, Sp4, and ER-α. The association of Sp3 and CREB was slightly increased by estrogen but not to a significant degree as compared with control DMEM (Figure 5c). These *in vivo *results complemented those obtained with DNA-pull down and electrophoretic mobility shift assay experiments and corroborated the role of Sp1, Sp4, and ER-α, and of CREB, respectively, in estrogen-regulated and basal BRCA1 transcription.

**Figure 6:**
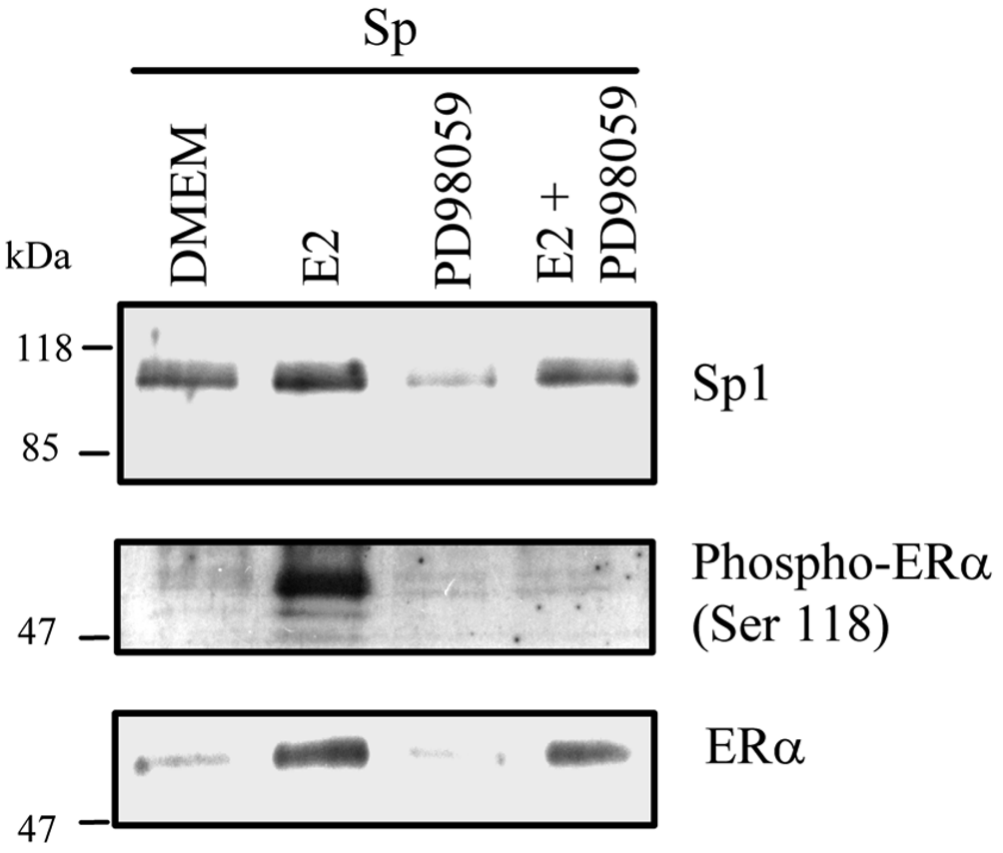
The MEK-1 inhibitor reduces the E2-induced binding of Sp1 and ER-α to the *BRCA1 *promoter. Nuclear protein extracts (200 μg) were obtained from MCF-7 cells cultured for 1.5 hours in Dulbecco's modified Eagle's medium containing 5% charcoal dextran-stripped fetal bovine serum containing vehicle (DMEM), or DMEM plus 10 nmol/l 17β-estradiol (E2), the MEK-1 (mitogen-activated protein kinase kinase-1) inhibitor PD98059, or E2 plus PD98059. DNA:protein complexes were generated by coincubation of nuclear extracts with the wild-type specificity protein (Sp)1/activator protein (AP)-1 oligonucleotide and separated by SDS-PAGE. The recruitment of Sp1, total ER-α, or phosphorylated ER-α was visualized by Western blotting with specific antibodies. Panels are representative of two independent experiments. Numbers indicate molecular weight markers (kDa).

Exposure to estrogen has been shown to activate signaling cascades, including the MAPK kinase (MEK)/MAPK phosphorylation pathway that leads to phosphorylation of ER-α and related coactivators [41]. Therefore, we examined whether the MEK/MAPK pathway modulated the binding of Sp1 and ER-α to the *BRCA1 *promoter regions comprising the Sp and AP-1 sequences. Compared with MCF-7 cells cultured in control vehicle (DMEM), the treatment with estrogen stimulated the binding of Sp1 and ER-α. This effect was counteracted by the cotreatment with estrogen plus the MEK1 kinase inhibitor PD98059 (Figure 6). The constitutive binding of Sp1 and ER-α to the BRCA1 oligonucleotide was not influenced by the treatment with the PD98059 compound alone. As a positive control, we measured the association of phosphorylated ER-α, whose binding to the BRCA1 oligonucleotide was reduced upon cotreatment with estrogen plus PD98059. These data suggested that the recruitment of Sp1 and ER-α to the region of the *BRCA1 *promoter comprising respectively the Sp and AP-1 binding elements was mediated through MEK-dependent activation of phosphorylation.

## Discussion

Understanding how estrogen regulates BRCA1 expression may contribute to clarifying the role played BRCA1 under normal physiological conditions and pathophysiological perturbations that predispose to breast cancer. Previous studies from our laboratory documented that estrogen stimulated BRCA1 expression by inducing the recruitment of an ER-α/p300 complex at an AP-1 site located close to the transcription start site of exon-1b [33]. The objective of this study was to define the role of Sp and CRE binding sites flanking the AP-1 element. The Sp-binding domain (5'-GGGGCGGAA-3') shared homology with the Sp1 consensus sequence (5'-[G/T]GGGCGG [G/A] [G/A]-3'). We found that mutation of the Sp site reduced basal and estrogen-dependent *BRCA1 *promoter activity in MCF-7 cells expressing endogenous ER-α. In transfected Hela cells expressing exogenous ER-α, the mutation of the Sp site led to a striking reduction in basal activity and reduced the estrogen activation. Cumulative data from DNA:protein binding and ChIP assays with BRCA1 oligonucleotides spanning the GC-rich and AP-1 domain suggested that estrogen stimulated the recruitment of Sp-1, Sp4, and ER-α but did not affect the constitutive occupancy by Sp-3. Therefore, the estrogen-dependent activation of the proximal *BRCA1 *promoter requires the intact Sp-binding site and appears to be mediated by increased occupancy of Sp1/Sp4 and ER-α as compared with Sp3.

Previous studies have documented that Sp3 can both enhance and inhibit transactivation. For example, the estrogen-dependent regulation of the *VEGFR2 *(vascular endothelial growth factor receptor 2) promoter was primarily regulated by ER-α/Sp3 and ER-α/Sp4, but not Sp1 [42]. Conversely, recent findings by the same group [43] documented a role for Sp1, Sp3, and Sp4 in regulating the *VEGFR1 *gene. Similarly, other reports corroborated a role for ER-α/Sp1 in estrogen-dependent activation of the interleukin-1α [44], insulin-like growth factor-1 [45], and cdc25A [46] gene promoter. Our results indicate that Sp1 and Sp4 play a specific role in estrogen-dependent regulation of BRCA1 transcription. Interestingly, mutation of the Sp site did not prevent the recruitment of the ER-α to BRCA1 oligonucleotides harboring the AP-1 domain, but it reduced transcriptional activity. These findings suggest that the estrogen-induced association of Sp1 and Sp4 at the GC-box may be functionally required for estrogen-mediated transactivation of the *BRCA1 *promoter. The observation that the Sp-binding site enhanced the recruitment of nuclear proteins to BRCA1 oligonucleotides comprising the AP-1 and CRE elements suggests the Sp site may increase the stability of the ER-α/p300 complex recruited at the adjacent AP-1 site. In electromobility shift assays, we observed constitutive recruitment of CREB to a half-CRE (5'-CGTAA-3') located just downstream from the AP-1 motif. However, the constitutive occupancy by CREB was not altered by the estrogen treatment or the presence of the upstream Sp-binding site.

**Figure 7:**
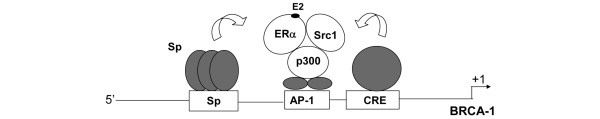
Proposed model for regulation of *BRCA1 *promoter activity. Results suggest that specificity proteins (Sps) contribute to basal and estrogen (E2)-dependent transactivation of the *BRCA1 *promoter. The Sp-binding region is a target site for multiple Sps, including Sp1, Sp3, and Sp4. However, our data document increased recruitment of Sp1 and Sp4, but not Sp3, in response to E2. We also document the constitutive presence of cAMP response element (CRE)-binding protein (CREB) at the CRE site. Arrows indicate potential interactions of Sp and CREB factors with a p300/estrogen receptor (ER)-α transcription complex bound to an activator protein (AP)-1 (Jun/Fos) domain [33] harbored in the *BRCA1 *promoter. The TRANSFAC/Match transcription factor database [56] was used to map the Sp, AP-1, and CRE sites.

One possible model that integrates the current results is depicted in Figure 7. Activation of BRCA1 transcription by estrogen may be dependent on the increased interaction between Sp1, Sp4, and ER-α bound respectively to the Sp and AP-1 elements located in the proximal region of the *BRCA1 *promoter. The occupancy by CREB at the half-CRE may serve as an ancillary factor that regulates constitutive activity. Interactions between ER-α, Sp1, and Sp4 may trigger the subsequent recruitment of cofactors such as p300, which can function as a histone acetyltransferase [4], thus leading to estrogen-dependent activation of the *BRCA1 *gene. This model finds support in the experimental evidence that mutation of the Sp or AP-1 [33] element prevented the estrogen-dependent activation of the *BRCA1 *promoter.

The role of Sp proteins has been proposed to be promoter selective and dependent on the presence of flanking sequences [42]. The Sp1 factor has been demonstrated to interact physically with the ER-α and ER-β [7], but only the ER-α/Sp1 interaction led to transcriptional activation of estrogen-responsive genes, such as *Hsp27 *[6], *cad *[47], and *cathepsin-D *[48]. Conversely, ER-α/Sp1 interactions played a minimal role in activation of *VEGFR2*, and ER-α/Sp3 and ER-α/Sp4 were critical factors [42]. In this study, we found that the treatment with estrogen increased the binding of the *BRCA1 *promoter by Sp1 and Sp4, but it did not alter the levels of bound Sp3. Similar to other genes involved in the oncogenic process [49,50], the relative occupancy ratio between Sp proteins may play a critical role in transcriptional regulation of the *BRCA1 *gene. For example, elevated Sp3 levels have been shown to promote pancreatic and breast cancer cell growth by inhibiting transcription of growth inhibitory genes including thosse encoding the cyclin-dependent kinase inhibitor p27 and the type II receptor for transforming growth factor-β [8,51]. Moreover, pancreatic cells transfected with small inhibitory RNA targeting Sp3 exhibited increased cell cycle distribution in G_0_/G_1 _and decreased distribution in S phase [8]. Conversely, Sp1 sites were required for activation of the p21 promoter [52]. Based on the observation that Sp1-mediated transcriptional activation can be repressed by Sp3 [53], a shift in the Sp1/Sp4 versus Sp3 binding ratio may alter normal estrogen regulation of the *BRCA1 *gene. The model depicted in Figure 7 of cooperative interactions between DNA-bound Sp proteins and ER-α recruited at an AP-1 site is similar to that reported for the E2F1 promoter, whose transactivation by the ER-α was found to be independent of direct interactions with promoter elements and was regulated by a multiprotein complex comprising ER/Sp1/NF-Y [54].

Regulation of *BRCA1 *by estrogen may require activation of the MAPK cascade. Estrogen binds to the ER-α and stimulates MAPK-mediated phosphorylation of MEK1/2, which in turn phosphorylates ERK1/2 (extracellular signal regulated kinase1/2) [55]. The activated ERK1/2 may add phosphate groups to ER-α and Sp and modulate protein-DNA or protein-protein interactions of these factors at target promoters. Our results document that activation of the MEK pathway plays a key role in the estrogen-regulated recruitment of Sp1 and phosphorylated ER-α to the *BRCA1 *promoter.

## Conclusion

We report that estrogen-dependent activation of BRCA1 transcription is mediated by a GC-rich region, which is a binding target for Sp proteins. The Sp-binding motif is located in close proximity to an AP-1 site, which is a target for an ER-α/p300 complex. Estrogen stimulates the recruitment of Sp1 and Sp4, but not Sp3, to the GC-rich region. We also provide evidence that the AP-1 site is flanked by a CRE-binding domain that regulates constitutive expression. The close proximity of the Sp, AP-1, and CRE-binding sites may be important for the recruitment of transcriptional complexes and regulation of basal and estrogen-dependent BRCA1 transcription. Finally, we provide evidence that the MAPK pathway regulates the association of Sp1 and ER-α with the *BRCA1 *promoter. Overall, these results offer new evidence for both genomic and nongenomic regulation of BRCA1 transcription activity by estrogen.

## Abbreviations

AP = activator protein; ChIP = chromatin immunoprecipitation; CRE = cAMP response element; CREB = CRE-binding protein; DMEM = Dulbecco's modified Eagle's medium; ER = estrogen receptor; ERE = estrogen response element; FBS = fetal bovine serum; MAPK = mitogen-activated protein kinase; MEK = MAPK kinase; PCR = poolymerase chain reaction; Sp = specificity protein.

## Competing interests

The authors declare that they have no competing interests.

## Authors' contributions

JKH, SCD, and DFR conceived the study. JKH conducted the mutational studies. SCD conducted the DNA-pull down assays, Western blot analyses, and ChIP studies. SSM conducted the binding and electromobility shift assays. MQK and JKH performed transfection studies. JKH, SCD, SSM, and DFR contributed to the drafting of the manuscript, which was approved by all authors.
